# Biochemical and Molecular Analysis of the Hb Lepore Boston Washington in a Syrian Homozygous Child

**DOI:** 10.1155/2017/1261972

**Published:** 2017-05-03

**Authors:** Monica Pirastru, Laura Manca, Sandro Trova, Paolo Mereu

**Affiliations:** ^1^Dipartimento di Scienze Biomediche, Università di Sassari, Sassari, Italy; ^2^School of Biosciences, University of Birmingham, Birmingham, UK

## Abstract

Hemoglobin (Hb) Lepore is composed of two normal *α* chains and two *δβ* fusion globins that arise from unequal crossover events between the *δ*- and *β*-globin genes. The Hb Lepore is widespread all over the world and in many ethnic groups. It includes some of the few clinically significant Hb variants that are associated with a *β*-thalassemia phenotype. Here, we describe the first occurrence of Hb Lepore Boston Washington in a Syrian individual. The patient, a 10-year-old child, shows severe anemia with a Hb level of 6.85 g/dL and typical thalassemic red cell indices. The diagnostic procedure implies hematological, biochemical, and molecular analysis, including multiplex ligation-dependent probe amplification (MLPA) assay, GAP-PCR, and DNA sequencing. This latter allowed us to define the correct structure of the hybrid *δβ*-globin gene. The knowledge of the spectrum of mutations associated with different geographical areas is the prerequisite to set up large-scale screening programs and be able to offer genetic counseling to couples at risk.

## 1. Introduction

Under the name of Hb Lepore, a small group of structurally abnormal Hbs that result from in-frame fusion between the 5′ end of the *δ*-globin gene and the 3′ end of the *β*-globin gene is defined, formed by unequal crossover or gene conversion events during meiosis. The high sequence homology between *δ*- and *β*-globin genes favors this type of recombination that results in the deletion of approximately 7.4 kb. The *δβ* hybrid globin chain contains the amino terminal sequence of *δ*-globin fused to the carboxyterminus of the *β*-chain. Three Hb Lepore variants were characterized several years ago [1–4], each with a different crossover breakpoint: Hb Lepore Boston Washington (*δ*87/*β*116), Hb Lepore Hollandia (*δ*22/*β*50), and Hb Lepore Baltimore (*δ*50/*β*86) (HbVar database: http://globin.cse.psu.edu). Two further *δβ*-Lepore variants, Hb Lepore-Leiden (complex rearrangement) [5] and Hb Lepore ARUP (*δ*31/*β*50) [6], were recently described with a single case report for each. Hb Lepore Boston Washington is the most common Lepore variant. It has been found with a low frequency in a variety of ethnic groups, mainly in Mediterranean countries [4, 5, 7–9].

In all Hb Lepore variants, the synthesis of the *δβ* hybrid chain is significantly lower than that of the *β*-chain, resulting in an overall reduction in non-*α*-globin chains [10]. The Hb Lepore carriers display a *β*-thalassemic phenotype with microcytosis and hypochromia. Hb analysis shows 5%–15% of Hb Lepore and increased HbF levels that in most cases do not exceed 5%. Severe clinical conditions similar to *β*-thalassemia major were observed in Hb Lepore homozygote subjects.

The coinheritance of Hb Lepore defects with *β*-thalassemic alleles results in variable clinical severity transfusion-dependent thalassemia depending on the different degree of globin imbalance and compensatory HbF production. The most seriously affected were patients from Yugoslavia, showing hemolytic anemia, dyserythropoiesis, hepatosplenomegaly, and skeletal malformations [5, 7, 11].

The Hb Lepore has also been found in compound heterozygotes for Hbs S, C, and E. The clinical phenotypes of these conditions are extremely variable but, overall, resemble those of Hbs S, C, or E/*β*-thalassemia compound state [10, 12, 13].

In this paper, we describe our experience with a challenging diagnosis of a Syrian 10-year-old refugee child referred to our laboratory for biochemical and DNA analysis.

## 2. Materials and Methods

Clinical and hematological data were obtained. Hb tetramers separation was performed by cation-exchange high-performance liquid chromatography (CE-HPLC) (VARIANT™; Bio-Rad Laboratories, Hercules, CA, USA) and by isoelectric focusing (IEF) in the 6.7–7.7 pH range. Constituent globin chains separation and quantitation were carried out by electrophoresis on 12% polyacrylamide gels containing 6 M urea and 2% Triton X-100 in 5% acetic acid (AUT-PAGE) [14] and reversed-phase high-performance liquid chromatography (RP-HPLC). With respect to the original procedure [15], the elution gradient has been modified as follows: 45-minute linear gradient from 51 to 59% developer B at the same 1.2 mL/min flow rate.

Genomic DNA was isolated from peripheral blood by a standard method.

Three primer pairs (*β*1-*β*2, *β*7-*β*8, and *β*9-*β*10) have been firstly used in an effort to amplify the *β*-globin gene (Tables 1 and 2).

Searching for deletions and gene fusions within the HBB cluster was subsequently performed by MLPA assay using SALSA MLPA probemix P102 HBB (MRC-Holland, Amsterdam, Netherlands). Ligation and amplification reactions were carried out on a GeneAmp® PCR System 2700 thermal cycler (Applied Biosystems, Foster City, CA, USA). MLPA products were separated by ABI PRISM 3130 Genetic Analyzer (Applied Biosystems, Foster City, CA, USA) and quantified as already described [17].

A GAP-PCR using the *δ*1-*β*2 primer pair (Tables 1 and 2) has been successfully performed to amplify the *δβ* fusion gene and to determine the deletional breakpoints.

All the amplified products were electrophoresed through 1–1.2% agarose, 1x TAE, and ethidium bromide stained gel at 7.5 volts/cm for 45 minutes in the presence of a molecular weight marker. DNA was recovered from agarose by means of the Montage Gel Extraction Kit (Merck Millipore, Darmstadt, Germany). The purified fragments were sequenced by terminator chemistry (BigDye Terminator v3.1 Cycle Sequencing Kit, Applied Biosystems, Foster City, CA, USA). Reaction mix was purified through the Sigma Spin Post-Reaction Clean-Up Columns (Sigma-Aldrich, St. Louis, MO, USA) and subjected to capillary electrophoresis on an ABI PRISM 3130 Genetic Analyzer (Applied Biosystems, Foster City, CA, USA). Another Plasmid Editor APE (http://biologylabs.utah.edu/jorgensen/wayned/ape/) was used to align the obtained sequences with the reference (GenBank accession number NG_000007.3).

## 3. Results

A blood sample from the 10-year-old Syrian refugee child was referred to our laboratory for diagnostic testing. Unfortunately, blood samples from her family were not available.

The patient has been transfused before her arrival in Sardinia, but no details were given about the amount, frequency, and regularity of transfusions. Upon arriving, the proposita presented with severe hypochromic microcytic anemia. Her hematology showed a Hb level of 6.85 g/dL (normal values (NV) for the local laboratory: 12–16) and typical thalassemic red cell indices: red cell count 3.12 × 10^6^/*μ*L (NV: 4.5–5.9), MCV 63.5 fL (NV: 80–96), MCH 21.9 pg (NV: 27–32), and RDW 34.9% (NV: 12–13.5).

**Figure 1:**
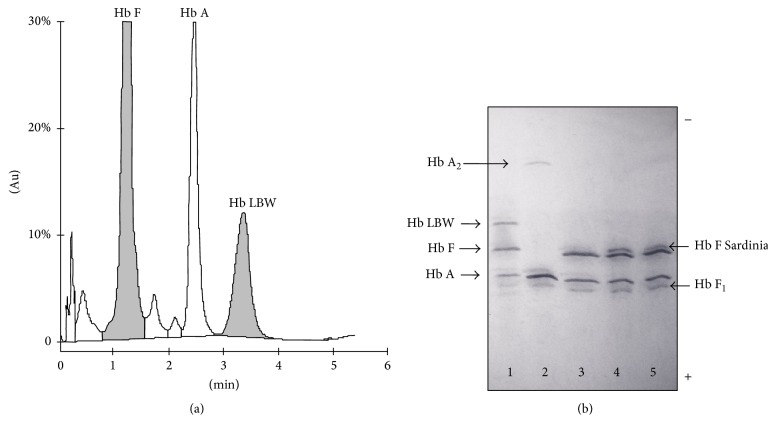
Cation-exchange HPLC of Hb tetramers showing Hb Lepore Boston Washington (LBW) eluting in the HbA_2_ window (a). Isoelectric focusing of native Hb tetramers. Lane 1: proband; lane 2: normal adult; lane 3: normal newborn; lanes 4 and 5: newborns homozygous for HbF-Sardinia [A*γ*75(E19) Ile → Thr; HBG1: c.227T>C] (b). For Hb F-Sardinia, see [16].

**Figure 2:**
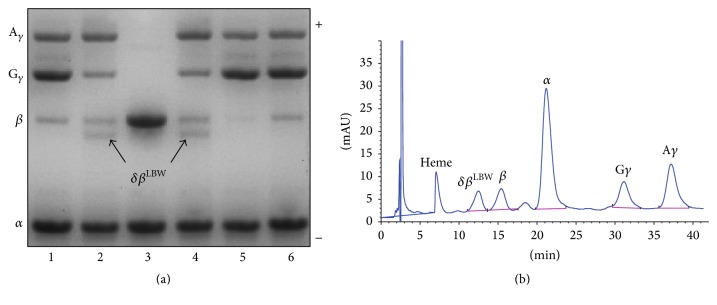
Electrophoresis of dissociated globin chains. Lanes 1, 5, and 6: normal newborns; lanes 2 and 4: proband; lane 3: normal adult (a). Reversed-phase HPLC of globin chains showing the abnormal fraction corresponding to the hybrid *δβ*-LBW chain (b).

The CE-HPLC profile showed a Hb variant which coeluted with HbA_2_ giving a combined level of 13.4% (Figure 1(a)). Hb quantification also revealed abnormal HbF and HbA levels of 57.2% and 19.7%, respectively. IEF confirmed a variant tetramer that migrated more slowly than HbF (Figure 1(b)). Characterization of the denatured globins, performed by AUT-PAGE and RP-HPLC, suggested a *β*-qualitative variant and highlighted a HbF composition of the adult type (Figures 2(a) and 2(b)) consisting of a *α*_2_^G^*γ*_2_ : *α*_2_^A^*γ*_2_ ratio of 38 : 62 [16, 18]. These results were compatible with compound heterozygosity for deletional *δβ*-thalassemia, causing the elevation of HbF, and a mutated *β*-allele codifying a structurally abnormal hyperunstable or hypoexpressed *β*-globin [19].

**Figure 3:**
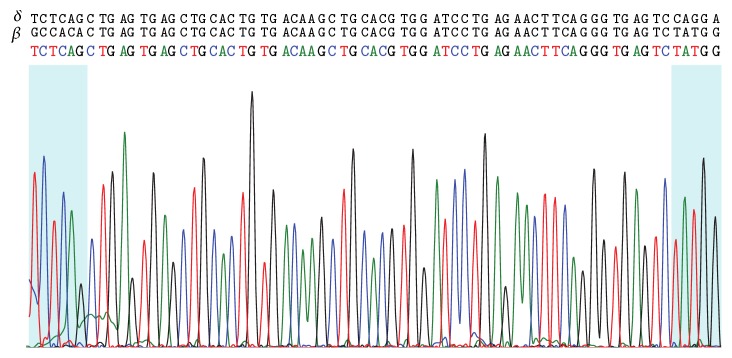
Nucleotide sequencing of the hybrid *δβ*-globin gene showing the crossover breakpoint that occurred between exon 2 and IVS 2. The 86 and 87 codons (left box) belong to the *δ*-gene, whereas the downstream region, starting from IVS 2 nt 8 (right box), belongs to the *β*-globin gene. The 58 bp region spanning from codon 88 to IVS 2 nt 7, in which the two genes share the same sequence, cannot be unequivocally attributed to any of them. The strings of capital letters above the base calling show the reference sequences of the *δ*- and *β*-globin genes (GenBank accession number NG_000007.3).

To define the *β*-genotype, PCR of the *β*-globin gene was carried out. The *β*7-*β*8 (exon 2 to IVS 2) and *β*9-*β*10 (IVS 2 to 3′ UTR) fragments were generated, whereas the PCR reaction using *β*1-*β*2 (5′ UTR to exon 2) primer pair repeatedly failed. Sequencing of the obtained products unexpectedly showed a specific *δ*-globin gene sequence from codon 66 to codon 87 and a specific *β*-globin gene sequence from IVS 2 nt 8 to the end (Figure 3). These results led to suspecting the variant to be the product of the *δβ*-Lepore fusion gene, where the large deletion would have approached each *δ*- and *β*-gene. Accordingly, the genomic regions recognized by *β*7 primer (which anneals both on *δ*- and on *β*-gene) and *β*8 (*β* specific reverse primer) get close enough to provide an unexpected GAP-PCR fragment. The deletion would also have removed the sequence where the *β*1 (*β* specific forward) primer anneals, preventing the generation of *β*1-*β*2 amplicon.

**Figure 4:**
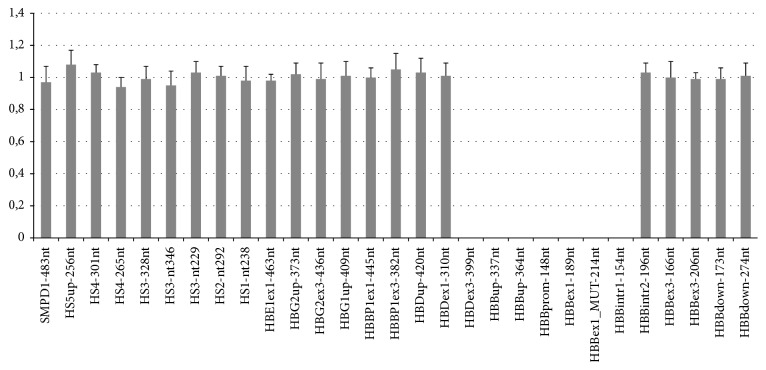
Multiplex ligation-dependent probe amplification performed using SALSA MLPA Kit P102 HBB. The horizontal axis shows the MLPA probes arranged according to chromosomal location. The vertical axis shows the normalized probe ratio. The complete absence of peaks corresponding to the probes HBDex3-399nt through HBBintr1-154nt indicates a homozygous deletion of the 3′ end of the *δ*-globin gene and 5′ end of the *β*-globin gene.

To test this hypothesis, the HBB cluster was screened using the MLPA assay. The MLPA results revealed the absence of peaks for the probes HBDex3-399nt (*δ*-gene, exon 3) through HBBintr1-154nt (*β*-gene, IVS 1) corresponding to a homozygous deletion of the 3′ end of the *δ*-globin gene and 5′ end of the *β*-globin gene (Figure 4).

To confirm the correct breakpoints, a GAP-PCR was carried out using *δ*1 (*δ* specific forward) and *β*2 (*β* specific reverse) primer pair. A *δβ* fusion product of 766 bp was amplified. No Hb Lepore amplicon was detected in the control samples (data not shown). As expected, sequencing showed that the in-frame fusion gene consists of an upstream region, spanning from 5′ UTR to codon 87 in exon 2, which belongs to the *δ*-gene, and in a downstream region, starting from IVS 2 nt 8, which belongs to the *β*-globin gene. A 58 bp region spanning from codon 88 to IVS 2 nt 7, in which the two genes share the same sequence, cannot be unequivocally attributed to any of them. These results are consistent with the hybrid *δβ*-globin gene corresponding to Hb Lepore Boston Washington (http://globin.cse.psu.edu/).

## 4. Discussion

There are a large number of studies on the clinical findings and hematological data in heterozygous state for Hb Lepore while there are relatively few chances to study the homozygous state. Clinical pictures similar to that of *β*-thalassemia major were observed in Hb Lepore homozygous individuals (a very rare combination), some of these probably only seen in children of consanguineous carriers. Besides, since some of these patients were transfusion-dependent, hematological and hemoglobin analytical data are often incomplete [5, 11]. Homozygotes for Hb Lepore display HbF together with 10%–30% Hb Lepore. Their clinical manifestations are rather variable, ranging from a picture of transfusion-dependent thalassemia major to a much milder thalassemia intermedia course.

In our laboratory, the characterization of mutant Hbs involves primarily electrophoreses under several experimental conditions and RP-HPLC analysis. This leads usually to an unambiguous result. However, although hematology and protein based Hb diagnostic studies may be sufficient for the identification of many hemoglobinopathies, a proper diagnosis of Hb Lepore, especially in compound or in homozygous state, may sometimes be challenging and require a high degree of clinical suspicion and correlation. For this reason, a definitive identification can be achieved only by DNA analysis.

The Hb profile of the Syrian child showed an anomalous fraction coeluting with HbA_2_ and a high level of HbF (57.2%). In addition, the HbA fraction amounted to only 20%, presumably being the residue of a previous transfusion. The presence of the abnormal Hb prompted us to suppose that it could be the result of a *β* mutant gene which expressed an unstable variant. Moreover, given the critical anemia of the proposita, we thought that this allele was coinherited with a thalassemic determinant associated with increased HbF level such as deletional *δβ*-thalassemia. This latter group of disorders is due to deletions of different sizes involving the *δ*- and *β*-genes and is characterized by the presence of substantial elevation of HbF in heterozygotes, as well as in homozygotes and compound heterozygotes [20]. Contrary to our presumptive hypothesis, DNA analyses, carried out using MLPA, GAP-PCR, and DNA sequencing, identified the patient as homozygous Hb Lepore Boston Washington. In such homozygous state, HbA and HbA_2_ are absent and the Hb is made up of HbF and Hb Lepore only. In the proband's CE-HPLC profile, the variant elutes in the HbA_2_ window and individually amounts to 13.4. The low level of the Hb Lepore depends both on the weak activity of the *δ* promoter and on the relative instability of the variant [21, 22]. For these reasons, the Hb Lepore is considered as a *β*-thalassemia-like mutant. Conversely, the counterpart anti-Lepore *βδ* fusion globin is not usually associated with hematological abnormalities. The rare interactions between the anti-Lepore Hb and *β*-thalassemia have been associated with very mild forms of *β*-thalassemia intermedia [21, 22].

GAP-PCR and DNA sequencing, in combination with the MLPA analysis, work as powerful screening tools for the detection of both known and unknown deletions in the *β*-globin gene cluster. Their detection is essential to differentiate the clinically severe form such as the homozygous state here described or *δβ*-thalassemia disorders from asymptomatic deletional conditions [23].

Our results highlight the importance of DNA analyses in defining the proper genotype in order to predict the severity of the disease, to ensure appropriate patient management, and to allow genetic counseling. In particular, the implementation of nucleotide sequencing analysis provides more detailed information about not only the breakpoint but also the molecular mechanism by which different fusion genes arise. In this sense, the case of the Lepore-ARUP (*δ*31/*β*50) variant is emblematic, which, despite showing the same amino acid sequence of the Lepore-Hollandia (*δ*22/*β*50), is expressed from a different DNA sequence and thus resulted from a different crossover event [6].

As a precaution, in countries where a high rate of *β*-thalassemic carriers is present and where large immigrant groups are living, it is important to include deletional types of *β*-thalassemia in the molecular screening for couples at risk.

## 5. Conclusions

To the best of our knowledge, this is the first study reporting the presence of the Hb Lepore Boston Washington in Syria. The challenging diagnosis included hematological, biochemical, and molecular analyses. Particularly, MLPA, GAP-PCR, and DNA sequencing, which represent elective methods for studying genomic rearrangements as deletions and duplications, allowed us to reach the correct diagnosis and to define the hybrid *δβ*-gene structure.

## Figures and Tables

**Table 1:** Tabulated details of PCR conditions.

Primer pair	Target region	MgCl_2_ (mM)	dNTPs (*μ*M)	Thermal conditions^*∗*^
*β*1-*β*2	HBB (5′ UTR to exon 2)	3.5	250	(94°C^1 min^-65°C^45 sec^-72°C^1 min^) × 35 cycles – 74°C^4 min^ × 1 cycle
*β*7-*β*8	HBB (exon 2 to IVS 2)	3.0	′′	(94°C^1 min^-60°C^1 min^-72°C^1 min^) × 35 cycles – 74°C^4 min^ × 1 cycle
*β*9-*β*10	HBB (IVS 2 to 3′ UTR)	1.5	′′	(94°C^1 min^-55°C^1 min^-72°C^1 min^) × 35 cycles – 74°C^4 min^ × 1 cycle

°*δ*1-*β*2	HBD 5′ UTR to HBB exon 2	′′	′′	(94°C^1 min^-60°C^1 min^-72°C^1 min^) × 35 cycles – 74°C^4 min^ × 1 cycle

^*∗*^Each thermal profile was preceded by denaturation at 94°C for 3 min.

°The  *δ*1-*β*2 primer pair is used in GAP-PCR. The distance between the primers is too great (>8 Kb) to amplify the normal allele and product is only obtained from the deleted one where *δβ* fusion is present.

**Table 2:** List of primers used to carry out PCR and sequencing reactions.

Primer pairs used for standard PCR and GAP-PCR			
Primer code	Sequence (5′ to 3′)	Nucleotide position (^#^NG_000007.3)	Product size
*β*1 (F)	GCCAAGGACAGGTACGGCTGTCATC	61997–62021	706 bp
*β*2 (R)	CCCTTCCTATGACATGAACTTAACCAT	62676–62702	
*β*7 (F)°	TCCTGATGCTGTTATGGGCAA	62469–62489	923 bp
*β*8 (R)	AAAAGCAGAATGGTAGCTGGA	63371–63391	
*β*9 (F)	AAAAACTTTACACAGTCTGCC	62935–62955	966 bp
*β*10 (R)	ATTAGCTGTTTGCAGCCTCA	63881–63900	

*δ*1 (F)	ATGCAGAGGAGAACAGGGTTT	54538–54558	
*β*2 (R)	CCCTTCCTATGACATGAACTTAACCAT	62676–62702	^*∗*^766 bp

^#^GenBank accession number.

°*β*7 also anneals to HBD gene at 55070–55090 nucleotide position.

^*∗*^The 766 bp fragment belongs to the *δβ* fusion gene.

## References

[1] Gerald P. S., Diamond L. K. (1958). The diagnosis of thalassemia trait by starch block electrophoresis of the hemoglobin. *Blood*.

[2] Baglioni C. (1962). The fusion of two peptide chains in hemoglobin Lepore and its interpretation as a genetic deletion. *Proceedings of the National Academy of Sciences of the United States of America*.

[3] Barnabas J., Muller C. J. (1962). Hæmoglobin-LeporeHollandia. *Nature*.

[4] Ostertag W., Smith E. W. (1969). Hemoglobin‐LeporeBaltimore, a Third Type of a *δβ* Crossover (*δ*50, *β*86). *European Journal of Biochemistry*.

[5] Harteveld C. L., Wijermans P. W., Arkesteijn S. G. J., Van Delft P., Kerkhoffs J.-L., Giordano P. C. (2008). Hb Lepore-Leiden: a new *δ*/*β* rearrangement associated with a *β*-thalassemia minor phenotype. *Hemoglobin*.

[6] Nussenzveig R. H., Vanhille D. L., Hussey D., Agarwal A. M. (2012). Development of a rapid multiplex PCR assay for identification of the three common hemoglobin-lepore variants (Boston-Washington, Baltimore, and Hollandia) and identification of a new lepore variant. *American Journal of Hematology*.

[7] Efremov D. G., Efremov G. D., Zisovski N. (1988). Variation in clinical severity among patients with Hb Lepore-Boston-*β*-thalassaemia is related to the type of *β*-thalassaemia. *British Journal of Haematology*.

[8] Ribeiro M. L., Cunha E., Gonçalves P. (1997). Hb Lepore-Baltimore (*δ*(68Leu)-*β*(84Thr)) and Hb Lepore-Washington-Boston (*δ*(87Gln)-*β*(IVS-II-8)) in Central Portugal and Spanish Alta Extremadura. *Human Genetics*.

[9] Ferrara M., Matarese S. M. R., Francese M. (2001). Hematological and molecular analysis of *β*-thalassemia and Hb Lepore in Campania, Italy. *Hemoglobin*.

[10] Huisman T. H. J. (1997). Compound heterozygosity for Hb S and the hybrid Hbs Lepore, P-nilotic, and Kenya; comparison of hematological and hemoglobin composition data. *Hemoglobin*.

[11] Weatherall D., Clegg J. (2001). *The Thalassaemia Syndromes*.

[12] Viprakasit V., Pung-Amritt P., Suwanthon L., Clark K., Tanphaichitr V. S. (2002). Complex interactions of *δβ* hybrid haemoglobin (Hb Lepore-Hollandia) Hb E (*β*26 g→a) and *α*+ thalassaemia in a Thai family. *European Journal of Haematology*.

[13] Fucharoen S., Weatherall D. J., Steinberg M. H., Forget B. G., Higgs D. R., Weatherall D. J. (2009). Hemoglobin E Disorders. *Disorders of Haemoglobin: Genetics, Pathophysiology and Clinical Management*.

[14] Pirastru M., Manca L., Di Suni M. P., Speziga S. M., Masala B. (2004). Hb F-Porto Torres [A*γ*75(E19)Ile→Thr, 136(H14)Ala→Ser]: a novel variant of the A*γ* chain having two substitutions, one being that of Hb F-Sardinia. *Hemoglobin*.

[15] Masala B., Manca L. (1990). High-performance liquid chromatography of globin chains in the identification of human globin gene abnormalities. *Biophysical Chemistry*.

[16] Manca L., Masala B. (2008). Disorders of the synthesis of human fetal hemoglobin. *IUBMB Life*.

[17] Trova S., Mereu P., Cocco E., Masala B., Manca L., Pirastru M. (2016). The new −474(C → T) substitution discovered in the *HBG2* promoter of a sardinian *δβ*-thalassemia carrier. *Acta Haematologica*.

[18] Huisman T. H. J., Harris H., Gravely M. (1977). The chemical heterogeneity of the fetal hemoglobin in normal newborn infants and in adults. *Molecular and Cellular Biochemistry*.

[19] Thein S. L., Wood W. G., Steinberg M. H., Forget B. G., Higgs R. D., Weatherall D. J. (2009). The molecular basis of *β* thalassemia, δβ thalassemia, and hereditary persistence of fetal hemoglobin. *Disorders of Haemoglobin: Genetics, Pathophysiology and Clinical Management*.

[20] Clark B. E., Thein S. L. (2004). Molecular diagnosis of haemoglobin disorders. *Clinical and Laboratory Haematology*.

[21] Cao A., Galanello R. (2010). Beta-thalassemia. *Genetics in Medicine*.

[22] Olivieri N. F., Weatherall D. J., Steinberg M. H., Forget B. G., Higgs R. D., Weatherall D. J. (2009). Clinical aspects of *β* thalassemia and related disorders. *Disorders of Haemoglobin: Genetics, Pathophysiology and Clinical Management*.

[23] Cui J., Azimi M., Adekile A. D., Al Awadhi H., Hoppe C. C. (2012). Detection of anti-lepore Hb P-nilotic by multiplex ligation-dependent probe amplification. *Hemoglobin*.

